# Supplementary material for: Kinetochore phosphatases suppress autonomous Polo-like kinase 1 activity to control the mitotic checkpoint
Source: J Cell Biol. 2020 Oct 30;219(12):e202002020. doi: 10.1083/jcb.202002020 (PMC7608062; doi:10.1083/jcb.202002020)
Supplement: Data S2 — shows the KNL1 orthologues from Fig. S8 that are not published in Kops et al. (2020), Tromer et al. (2015), or van Hooff et al. (2017). [file JCB_202002020_DataS2.docx]

>ACAR

MDRSYSETNEERDFTDGTIKRRISSILKAPRTPLQNLGSGNELIQDYNIEKRQRNSRRVSFADTIRVFPPDPQIIVELKQAVSETAGEPRVQDFFHKNEDPEVVSCEITGMSTLLHAPIQTCLQQTECCDAETGQAWNKMDRTVIFSEENEMDMTSGHTIVITHGIRIREETNNTRKINFKSFSAGKSDKETSQMNECHLFSGPAKMNDTSLSQQQLNTENPPKINFSDFLNSLKSTVHSLPTGVMERNVGYSHFHEEDCNTTTASGIICNIQNPITSRLSNKSTVTLPSKQVGCEQENTSIITTNMENVLPPGTSSLNMADSQERDWSASNNMDSGGLKTSRNQFGVQEDQRLCVNELTMNDIESIADKKHTEIKEDASFSYYKTSIMPRSIDSVTSMPVFNVDRTSVFRDFSADMELTRNCTGLAWEENFKGIGDPFAQNFGKQGNDVSRLMEETIFQEEHMDITNNQIPSHSYVHKTKHSALNERPGPLQEVTYNKMGLQNSRILGDPQTPFLPETNYCPLGQVTKSFGSQTVSPFLSSTHDIMQQAHTKAGENEMKRKSRLPNNMSLISNDQTARFHFSENMDITEPITYLENSLNAHGFLAVPQQGLESDQKNVIGLNSNKTTVFSLNEDNEMDITRSHRVPVNYDMQPCQKTLQACSIQSAVESVYMCNDYMDETGMINQFIENSTQKQSNESRRRTLTGQLKDRTVVFSLDENEMEMTKSHTVTLKRDVVAKDIDIPLSSALFSNKTAKLTVGEDMKITRPVEYVPESLQNTSKKNVMKSASEKTRPALTESNDMELTKRLKVAVDGTPQFSLIPSEKNSMSIHSSDMKISQSVHFVPENKTMLFMHNNDMEITKPMTSTSLKEAKVKDLEVEKEETIKVILQGRVKKWTDTYLYDNCEMEITKNHTVPVNHDILQQVKETPQELASASVGKTSIFAHLSDAKTQSKTFVPADKTFTCNNDMEIIKPVSDSRFTALSPKEKEIRKTTLLGLNKDDTTVFSQHDAEMENTKCHTVRVTHDVVSQCERAPQVSFHQNNMNTTGSHTVTKEMMADDGRVNKKHTITLDYKTDLPCKTTLQAESTFPVSTTCTVTSFHSTKVSSLPCNATYNGNLEESGKKEMPDKNIKQNMNKALSHTYVEKIPDVDVTENQVVSIYGSNNDVPDAEKEILNPFTKSKEKAAVSSDGNNMEITQNYKTGIEFMHINDSNECHPNSEAKFGSRFSALVNDYESKLEIAQTEKNQFEAPASVMLPMCLNTELKPKDYLQIKENLPALCNVNTFSNELTLHIVSNEGNGFMLPTTEPILSKETSNGLNSKKMPILVNEIEILKNNLNKSQSEQEASSSGQTDPLPFGKEYRKETREWSSITKDCQNNLESPLDNTFSSEKELDILSELSGVDSSCPKTKDTRKKSELVLLSQDVASTLRDVLPKLTTKLDSSLNMKEDPKKELDTVTSTEWTNVGFTERASVNVPLHTESYNITKLPLGIFPPKLPNKRKSTVSSTESATAGLEKRKQTQDSEVSLVIKRLSDKLAQNPNLSCYINEELLPAYEEEIDSNEALSCDIPERLYDVGKDEGIAGNEDSLSERFETNKRQRALNQGDEELQKEKKFKGDEGWNDSGDFKQPICSTATAYLQGAAQEGKNVPELITVNLEKTQSSNSSSLDSVQQNSEMETQLLVDSICEQNLQEKFQEGTITVREFFTLLEVHVLIQKPRQSLLPPKYEINTTPNLEDEIINQYVYHPKLLAYGEDCQALCKTVDELKVNANNQDKLLVNVHKSLWEVMRTCSDKELKVFGAELNKMKSCFTKKSKVLAHKAKAKLYAKLVQNAELQWEKLQSRLAKIDELLEEMDSCINDLETEAAALKETELDVNDVVSEYESKVRDTERELEELRVQEEALQRNQSNLRDKKQHNDSEIIHLQDYVKSYQEITEKYNFSEWVMKEWNDHQAVLTFLYDSIELIVRLECPVDGAGYKIVSVNFESLLDDDRALASSKLVHRLVFQFINSQSLQLEKCLTMHHLSQILHHVSLVVSRCQLLGEEIEFLDRWGGKFYLLKTEVNDTKVKFLFSSSVACGSFEVEFSLSANYPASPVAFTIQKCTGNLSQEDISVILSNVPVGANYLRRIVKEISHNVLQCSPSTIQKQQRVAVR

> ACEY

MDPPSRKSRRSSILKIRQTEVVLESTAVEDDQKQVLKRRVSFHNVKTVQNFEKDNLNLLDGSPFREKIQETMSSDGILTPGRSQVTLSTPSTATSNTENTNFIEDTMAAFEGDGPRQRLSYDEVTSYSAADMSLSDTTVTDATEAVFNATQALQLDDHSAVDMCISESTSAGDDGGLPNVNSNNTKYSSVDMSFSTTMDCSSRSSDDTLAALDVTCGNQKISANRIKGRDLDVSSKPSCDLSKRELMMDETMMAMNLKPGQSVSMFRTPAHSSHDKSSVSTYNDDTALAFEEATAVLTRGDDRENEMKLSTTISDSTISGDTMAVFETVERDKESAASGSSDMDITCVTTSASVLESHSPPLYTAVASEEKEEPGNATFVVEHRSPIQSLAKLPEEHIRCEQAVNDTVTLMNSEKSCRTEAESSSISANDDHGLAFTQAAGDYEESIHEQSVCRDATILISRDCDSSDVKASNLCSSHSTLLKSHLESPGVVDRKRQKRFNDKSSETTEGEELEEQRRADVSCYHATPAAVDAVFEDKGACGTVLLSNEDGTAGESMDVVQDHTAISKADCTAICDEKMDITQLSNEVIGTVPCNGDFNRTALMCDTLRTLAEQIEDEEIGPNQHNATENCSILSALNESNIARRDISLLQPGSRFRLSTSSRDDTVLSTKSIRDETISISHRMMFDSHALQVMYANPADDQPEGNLVREQIVEELTKKLVETRQRSECDFEALFPKLQARDAAKALAVKNMNPRALSIDEADLLQSARLRAEIEWANIRAEVATQAISGIEQCLAEDAPLLKQLCEDAQLCERLDELEKEVKDLENSIGGAPSPQEVAEILDSYERAVREEEDLDSRILDKEIESYREHIGEERSPRSRFKATDPPVSFFVKFFFLVNRHNSTHSGAYSQSVSK

>OTAU

MREGKHTPGKRRSSIGRVGTPNSADRTGTFTFNASQNMDDVTAEVPTMGALLDAEVTENITGTVPSLANLLSAVESPLGYRPTPPPRVRPDFTEDITGGVPSLAALADEDEENAATASGRSVAHDGEMDMDTGNTTDGLPAFDPLDDATQRAVDQAHASALDLANTVMMDEATLVFSVGAPTPVAAPTPSLTFAFDDKKDTRASEGAPPASANTRPFNLTDSTNLDLEGQAKDGMGTDTFNYIYGGNSNATATGMLAAHQPTVDFPTAEVAGLNAQGSVDATPANVGGVVATPNTDGSIASSHDGFTPGDESLRMAELLRQKKHGDEALMSMRKVYQDRTTPMDRRYGAPPPMHAFTPSRTLTMGGTNFMNFAGTPGAAMHNPLFGTPGGHTGTMSGLPLIPDALMSSKPIDLETFFHACEVSFMEAKNMGRRSSIALGGLASAPPPETQPEALRLCCLTAPLIEALEGFHTELQERMEQLTSETDKLRTTVEATQPPLLRYASSDDPAHLNELRRAGKALKKECQLASKERFAQQKMETEQLVSESLNFHSEILVKSSQSIANSRAIAAEAISSANGHIIKLRAQRAASSRRADAAKSASATKEQVLEALAIKRAALAAARRALTASDERVEQMKGRKLVIKGERERLQEELEAMREAQDAAADTTVNIKSFGGVDVDDTPSRLRRASQVRLRSKAKHAEGASEELGVLSGLAPWRLEGVGGPAGAELTIRVGSLFQVTLDASTGAGRVTVIDGPEMTPKGGRQFAAALAGAPIAWGEDAVPAGGVAGVLQELAPKLARAERVLDETEGCRNEFPRLNRINCSATGVLELTFVDFVNERLFSVKLNMMGGAYPYGSLSPEVTVQLTGKKGVSELPSIDAIRRAVNAVPVGPRRLTNVCRILDSIVSTGKNALTSFATMPAPAPRRPSVAPSLLYAR

>MPOL

METPPFEQKSYDETTTVFGIDEDGSVVKRRRKRRSLGRRVSFAEAPAYHIFTRDDEYVSPSHDAQDAQGSAPQHVPKRRSPRLASKMPAAGGDDKENRRPVLLNEVEDCKGRRSTKPLSNLEQTTGSPASRPPSWHPAKNTSSLSRDDGEICDFVDIQNCALQQKNNVFDEDVTLDSTTFTFKLSQLQALKSQADGDQDKLFEPAPSTKRSYGRKTRGESPGPGSGENDTSMSFTMIQPIIRQKLGNLHSGHVDGSTDMSITMQVSRKHSSFLGTDVSKQDEVIEIMKAYLSEDNGERSPVGAPLSDLNLPKSRKTLPTTKDTQVKVDKDSRVDKEIVVLTTASGTGGTESNVTKETYAEFQKESVTLAAIIEEGGSGNLIAPVIPAGCGRVIPTETASRHEETENIFLNPAVSVEYEKDDRGPRGPNREIEATLTMDGSVSGTYVLEPGGHRGSARRSDQSKNSTTTDNQEPSQHNGNAQNLQFAILSHEETLLVPGNEERGLIATGSEEHGKENTDPTSREKKILAFTEETAKSLLTRGDDASTATTPANTTDATVTNTTDFRGQNDRLFAADLSEGLERVNRGLSATCQREEATVTMDMSISPTSTSLPHVECKRPALELERSITLDIPSLQDLVQEEEDEDMVTDMKMDTEIVCSRPTVTSNHCNSATRENQVVPGFGRRKIQGEEVFLMDNSLNRPTCMRERVSEMSRDELDHDRSITMGIPRLADIIREEEDYTHIEGQKKAPSNVVFPSLLHEVRNVPSSSDGDFSGSITAQVPRCSDLLAADSVSPDRAPAWNKSRRSSWVAPLPSSLRSSELGHAEGGGNICVTPCSDSGMEIVDDCGEPTLYSREVASLQASRGVQVVNIEASSAQVLGKGGEMHINLQNLTPTGDSGESMDIVGDTTDPMLLSSTVRLAELSHQSENTTLADCDEQMVIVDSTRPPVPSGIAQILKVSERPRDRNSDRSEQTTDIVPEAADTAPHFGPVKRAKFSMDVENRTSTDGSEDNMSMVEDLPKLNSTVELSSKRIGSISRFGEPQQILAPALYLSPVKQSHSEAKVPNYQNYTPSPSPRTYDAKELINSVLQSSARLASVGTVDKTFTFTCQTTEVQQHSLRKLQERLTLQLHSAKKLQLSAGNQSSDCTPTIRYKGAEQACSLADQSDCQHGKDGDTISVDDFFKLTEVNFCAKRESSGCASKDKFSIQAYQVNTIDAALKHFFLVQPRIGHMMEACSLVQDDLEKIRTKEARLEDELTASNPSLFSRFQKVGPGEKQQIKGRIHRLQTKCRLVEKRHWLNFRLGLEKDWNVKLLQSKSELQLHVEELRNGNSFLEENYQQILHLQARIPTNEFEVEEYPSDKTAPKAVEEKARNKCALDKIKYLKEQLVDVNSSAKTKLEKLLNRTKVTEEALRTSKERLAFSQCRLHDLQTKLAEHVRMGHSPAAGLQIAARPEFYVKEMSNKVSLLKALQGGWQVAVVGQDIVELSYTRKYLLDTVGLVKRRLVVAKTAGTSHVIESTWDVDFAGVELIFSRINAVEMFKQTFTDRPDVRKSQSLRSYIQATRFLVSNLLVLMEEIGECQKVTKAFARGVLVVPRFLLNDDEQMQLEIKFMDCLLSVNFILTLGMTYAQRDGYPFGAMPAKVDMVMCPRSLSHKLNSAALEGAIVGVEPGFHRVKRICAAMGSFLATLRDIRVD

>WMEL

METRRKSTTFLEKDKDKDKENLKLKESKKSRSKSLGGHHLPRDGDGDGDGDEDDDDLTPSKLARRKAKPRRSILKPQPPPSEDEDEERELELQSAKKAPRKSEASRRVSFASHCFIRLFEDEDGNKQASSSPRKSPSPRKPKPSPQKSPSKQSNDSDTSDMSIASQDVTFAFKNAIERKRPLDGSDTESDDEPTQSQEAQDDNNDDPTMQMDLTEAVGGFINKSPQKSPPKSPHRSPHKSPHKSPHKSPHKSPHKSPHKSPNTSTRKSPRLSAHNQSDDVKIEEPPLEMPKFTLSPTASEKANDSSSSDNDEDEADTTMDFTVSMSQTGVPKLPQFETGRRRSSLDRRSSVIKQNSGSPEGPSVVSMDVDNSSSSDDEENDQQMLLQPPPPAQIQTSQQSHSPSDSDSDSDGNGDDMDLTKAVTTQINTGDVDNNAQPQQEGDASVTMDMTQAIGGLLQNDKSQTEINDKSDIKEDQSDDDNSIASNNDQSMTMEMTQAVGGFTNFPTIPEEESSNSDNDNDVDATMTMEITRPIGKGILINDETRRLRKSLGPRMSRVSFAPSVKASPPKASNGPRPSLKSALKSSSSFEQSQSPKKAISPKGSPKDKTGKTRSSFSAPTQSSMLKKSSVRHSLGGALGGTTSSPLRSALKNASARSPGRAKKRASFAGDLRQENVEPSKPMSPLVQRLQLASPLRPPPFKLDVNGEKDEDDKSNKEEGDKHTKKPRRRSSLALSTHALEATPDTLASEKAKEEREKKDKERVYEKEVEEERKRKSTEEEKRIKEAEENQKTAADEVSKSNIDISHVTDMPQQSHSTNDNSDAVDKSISGEKSQVQAPQLGTPTRRRSLHASQMTPSKLRNSIIASQTNSPNSTPKRQSLRLASSVTTPQKEAFAEEKAEPVAPPARDEFAAGPETEKMIEGLMQEDEDVDNESSVEAPVQISLNDFLDLTDMKFLDGISTIKRRSTVGVGGLGGLKQQSQKDPTSLDYLRAHNVTGPKLEMMYWCCHELKRYISEGIEALNSYEREVDNENPPVIVDYLLASEEMRIRIEAQLKIVKNNSRLDAKRVWYGWRKELVHGHSESINDSFKSLEKDIGVLKETRGALKEKLPELRSLREKLEVELRREREIVRDIASCDKEEVEGLREAIAEQAQPLELYKAEISSNNEELTRLRTRLEAKKETLSNNRLAIENNRNEWDMVRCLTKAEAMKRRREYESLQSLHSWHIQHLSQSHKKFNYAEDIEINITDMGVELILLPPKKGELGPLLEYLLDCLRGQLTEEGISHTTELSYTLQRVSTLWTKVKKVHSEFKNLAQMYRLLFTVSDEGDLVVKLSILKRLQKLQFEVTIGSDLLVDLSSALRKARIKTDVIIGEDVNVETITQSLMAKLSNTDDKSIVEAFDGIVI

>CNEO

MSLAARSPRSRKSLSAFDTNISANFTLPVTNKGKKRVVASMDGGALRDMDAASIRADSFNMKSNPRRDVQPRKSILKSFSAPYITHQAEDETADYSASHQYAHTVAFGPLSSNVRSDPTSRNSRQSLGGRRVSFAPNAHVRMFERQVTKNPRASFGFSFTETSTPSLKSNHSRRSSILNIGSVSKPNIFAPSIFQGEGEAQGEESMEIEDDKELSGFDTSERDEAQQEHGHDGGAEGEESMEMEEEDMDITQHIYGGIVRRSSIAPTVAAPSELDAGADTEEESDNGQEHSHLEDEEKTMDFTIAVGGLLPHEAPATATRNRNSIGYSFPNPDGPALPNLMPGQAEDHEVEYPMDETEAYGAIIGQDISFSSGSEDTMGSRNGDKTMTFTYNHHIALPTQIDGDDSMDMITSAGGIISLPPVSPRLSVPNNTRPINGTPSFARPTVSSAQKTTATKRNVFAPSPSPLKSTTPRKSGIQTAAEVAKRLSFGSVTSSGGKKRVREDSQEAEGSMKKSRIEAAAEEVFGTPHHVSPFNKPEEAEPSTLIVQSTTPPSDPPQGTVMSTRRSSLGTAMRLSLPPQRSLAPPQQEAEEQNKKNVTEPQQRMHEQPQDISLAAFLEIAGVQFMEGLPGLNRKRSSVAKEILGQSYANERDFALHEYTEAQVNSIFLNMYTWASNKLFQDIQTGDEELTAVAARCDIDSPPVIQEYLAASDEDKQLFEMTFKSFKTNTHLKAKEMWYDWMWQLLETIKPDVETVLMGMKEDKKRLTAFEEQAVVLLPQLRARKIEVESKLVEERKAVVEIESCDQAELAAYKEAIAEQSAQITNFSTEVADLKDELATLTGKLEELNAKKHEYETAIAHAKGQCDQFTRSDAIRLQEESISLQHLHMWHPTKILPERMELTYDAEILLSINCSNYIPDISSATLEYLSERMQSSKRKCSGIRGESPSRCLFEVFKGAVGDMIKHKTWDLPSFVQQTGLLWSNAQRIRAELRYIEFHHPLFITYNSSTFLMSVSASIMIPQAKSKVLVWFDVDKSVVHGFPGSLGGVVVSVKSVYGQADVNLLEQTARQTIEMSRPEACLGTFLQVCVEVGARYSS

>EGLA

MAVSAGTGRSPRSARRKTITGTGEGAPHLQHAKKRAHSLAPRTSDLAPLRGILKPRNTDFGSADNTRSLDTTASTDFVTAVQDNTTSRKSFGPRRVSFAAHAHVRTFETDKREYAGSTSSPTPSPEQQRQPQPPATPIDDEDMSETDEDEPPPGVTAASPQHTGSLPPQRRPSLPHYPDDDGEGEASMEVDDDDAVGNEAIPQEAPPDEFDDDDMSDDSVYGDGILARRRSSTAPQAGRRSSTRRRSSIAPSRASTGGESMEFTMPLGRTLYETKPASEVMLQLQKATHSGRVSIVPPDDEDNHVADDGDVLGPLAVPQNQDASFSTDGDMSLDGGDADNRTLNLTTASSRRSSAMSLDSTGPGTPRIAPPQVASTPAFQLQPRAPTTPAQPPLATSTTPASQPATFILRPPPPNRAPSPATPRRSASPTKPTSAARPTFTAAFAPKTQVSPRKRAASVSASDLASPAKRQAVASGNASTPVRAQTEPPSSSPSKGAGTHVKRPPVRRSSSFNRAAQQQQQRDQPEKAPTPPLAPSSAALSENVPPPSQSTTPLAAPAPTSAFTFTSRAPTGPIEPSSSPIRHNVNANANDFSGSSSPAPAAPIYPSFNDFRPRALSMIPEMPTPTATISTARMYPSLAGPGSPSCEREEMRQARASPTPSRGSPMPRRSVAAEVEPSPVGGGLASQLARLTTSDDPRTQEWAEGVDADGQPDELQMTMDEFFAVTGIKFMDEFTCPRYSTALGLMNAPPENDDQVYELEQYFKALAVDVQQLETYQWTTNFLQGWLATSKANYAEAEQNVLSNPPAIFQAFVFADEEQRASMMHQLKIIKTNSLALAKAEWYAWKSDWIADLRSKADVCLAELDEDEGKLRDAARRVNDRLPEIRAMRDRVMRELAEERATVAEIETCDQDHLASLKAEIDVQAAEIERQKNEVADQETTVAHMHEKLAEQEHERREVISAIEDADRQSAHQRGCTREGLHAQLDELDAMQALHGWRIARLSPALVELVYADKFTVRIPCREWDALGVEVDVVHGAEGKMKKPGWRKDKYPDATAFMLQALQRQATARIRAHRRYSYGQVVRYIDTMWHAFACVRREIDLLAVRFPVQVTTDTASASLRIAANILVEKLESKAVASFVFNDDALLQWPLSVENVRVDAVIKYGDIQRDDVRMAVQERLVHTKPTDHYGHLLECCLEVVARYD

>RSOL

MHRRQSIAVPSHSGTNKENGSRVAKFRRTHSIGGPIDALKGNIGADGLSPRRRKRRSMAPRKSILKMHNEEEETMEMTTVVGGINSEMTMGRKSLARRVSFAAMAQVRMFEPTVNSNATASSPSHSSSPAAPQTSNDAPTPQKSPVATRRANSEEVGEASMELDDESMEGGDSFAIGDDEPQQEFDGGEGAGEDSMELTDTYQMGTPSRRRSSAPLGPLPPLDRSVERHKHRATDDSHVEEPAGDKDSGEYLVKTGKSIVPRRKSEAWAELQSLTGAAGSDMDEDSTGSTMNSDTGGGFIPASQESSGNAISSQGTVFSSQGTAFSSQTTTSSQGVGDMGLDDALSRLRAARQSMGAGAADMSIDDDDDRSSSSGDFEGGYDDDDRTMDVTAVTGGLRFSGDDDDEDEAEGEKAPDSAPQATEPTRKPTPTFKFSSQPEITSGTAAAAQASAPNLPTFAIDPPSPAPRVSPPVAPFTFSVPPPSKPVEKPVVASKSTSVVPAFSFDLTSSPAAPTPAFSLEAAPSLSASTKATPAETTKPTPRAKLSKLTPPSDAQPAPRSPITFEVSQSPVRAPPVTWDSHGPSPFEFKLGTPRRASGVNSALKVNATPNRAVTPVPVGTPKVVDTPARATSAPPTPKRPREETEEEPVAKRVALEAAAKKTSDVPAKPPRRRSFAPRMSVMGRGGVRLSIIPAEHEPEPEPVQLPTPDSAEPPASVFVQPPLPVRELSPVEPPRPATPEPQQPPLPLGHASPRPGAAALEEIRISSPLRSRVSMAGPNAPPKSPAQWRTGGAQNEVELDDMPTISASDFLRMTRISFMDGLTVKRRSTIGLGVLSRRKSDKDMVAGVADYVTAMTIGVPQLETYNYAAKELKEYISNGKKAIKILEEDLDANNPYLFKEYLASGEEDRRAIEETLGGHKEAMRMRSKMSWYKWRHNFVSDMQVAADGETELLQQDLDSLRAIGTRLTEPIPSLREQHAKLKAQLAAERAAVEAAKDCDPEIMSELKVGISEQSAQIEGYKADIQSSTAKLEKLKVKLEEAETDKRALQDQIRVHQEKLDALHPTVEIVNLREEFDKLQRLHLWQAVKLEEKFIELRYDDHYRVQMECVAFKPIPSGCRILVMPREGKQADEFPVLSELVLDLAQAMIRQATNLNLKKVVRMLGRLWTSVSHLRCNLRLLAMKYPVTIIRADCGFGFKATARVRFPGAKGLAKVMFIAEEQHIRDWGRQLNTLGVDADVVFGKLDRNLLVEVIRERLSDAIIEESYGLLLDACADAIACTEE

>ABIS

MAVLKNDAPKRRRSIAAPTQNQKSLVAPVSKRRAHSIVPGERLSILAKARRSLVPRKSILKASSIINTEEQTQQSSQSSNSQSSLPDDSNVTESMDLTLEYRARIHDNASRKSLGRRVSFAEHAQVRLFQTPNHDNTNSTGSPQSSPIPSSPEADVLPTLSNENDYPRGQRRSSVRYSMAGSEDMDMSSDNLGAFLDSGEGSALMGEELDLDGNDNMDATEALRGNLPRRRSLSGRQPFAPIRPRDSIMFPEDGTIPFDNDDDDDDDDGNSAQSMLEEDSQVQSEAENSQEGMEFTVPMGQSLKPPATEDPVWLALRQVTHSGDTPHEPEASSEDDIQVAGSQQGMELNDAMARLMRARDSLGEHTEDIIEDMEITSVNGNFAARDDSSSSSDDTPNDDFGNGDETLNISKIVGRLSLGRMSLGFQDTTMDESGIYESTIPLSSSTPQQSLAQPPPEKDRLTPDLPSDLPDFPETPEPERVEAPRPSVFLPPTEETNVPPPLPPITPLTTPAIATEQPPKSPVFKLIPPPPASDSAVLQPPKPHARHISPAKPKPKPSFSAAFAPPVAKPSPKKLSSAQSTPVNGTTNKRRFSVMQDGAPDTGRSSPAKRPTLGPKSIAGPSPNQRSAPSPGKNTPRKGSHAPSAAPKRQSGYFARRKSLGNALIVPTDGRGESHISTPSSLRENAGRARASLGSTLPEDWTRLDRNDLALTLPTIVPPTGKSQQEEARQEPVPLPISGPIDPPIPVSVASPEPAIIPHGRTPPVTGDREDENNVREASNNVGEQPLQTSPAEDIPSISIDQFFSMTGIKFMDDLTTPRRSVYPHTGSRKSRNPTDIPLSEYYPTMGIDVPQLGLFTKVSKDLEGWMARSKADFAQAEEEAAKVTPELFVEYMRADEEGQAELLHQLNFIRTNARGQAKSDWYDWKLKWIEGLQDTAEQTFTDLQSDAKSLEPIMNSGEELVFALEKECDELLQLLEHEQEEVAEIEASDQNYLNDLKDSIAEQNFEVEALRAELSEQTEQLNYLRGRLQEIAISKQEEANAYAKAQHFLEMKENSTRTEVFRLRGELEALENFHQFRVTRVDESLFEYVHASRYKVSIPCNNYLPITSNAEISHVVDAKAIKDDFPQYSSIVLQAAKALVHRLYPKSCKMVGPRTTPLSSILTIPQILQMLSEYWSCCSQLRAQLRLVSIKYPIEFLPMDLDGMPGFKVKVKVIIPSKKAKVFVIFNFTCDIFSKWPMSVSHLTSDVTVAYGPVEQSTLAEAVSERLSQATATENYACLLDACIEAQEIYNQ

>GLUX

MVVSIESRRKSIAVAQEGRSTLPKRKRAYSLGPSKLSPLSRSRLSIGNVKGILKSRPSSASSSQGTNSPSSQQSRPQSQSFADENNATISMDITQDYQAPINDNFSRKSMGRRVSFRDSVHVRFFNKDKGNDTGTSASSQESPGGSSSGAPSSDDDPIPAIPHVFNDENAYPGANPRRLSTRKSIAASEDMDMTSTGLGAFINGGDNASALLDEDMGLDDENSDMDITQSFTNEFRRRSSVGVSRVPLSQISSNPPQDQETSFTSEHSYTSEGDNSEAMEITAPLGKSLRPAHQDEAWLALVKATHSGDASMATSDDTEGDGMDDEDVEAMIARDAERRYTVNFNDVSDESMSEASFDDAGNQTLNLSKVMGRPSYGMNMGRPSVASSMDESEIYGEIVGGSASTPPPNPAQPQPNPVEATQEQPRSVSPTRPSSRVFSAPTSNPPAPTPLVFKPPPVTSPKRTTTSTPRSPTKSPAKPLLKSFSAAFAPPVARPTPKRSAETTDEALPSATKKRERAISAPSPAGIEVPDQPSPAKRQALEAKWSNKVAPSATSQPVSASMAAEKNRPLSPSKRIPFESATLERPTNIRRPSGYYARRKSLGAGLASSSSVSQEQGDASSVAVQSSPKKTATLGSRRASVSSGSSKAWARFDRTTVLPTGYSNGEGKTASIAEREEELGNTGNSVVVSPSPEIIAPRSIPASAPPLESMEIEAGPSVSKTPVVDLSAILETSGFGDEEEEREEEEEIKSGPTGMNMAATEQWRDTIPEDGYAVDEGPPISIEQFFELTGIKFMDLTAPRRRSTHASQIPPSEARDPTKIPLAEYAVAMAIDVPQLDHYSRVSRDLEGWMEQSKVEFEQMEEEAAKMTPELFAEYSQANPDEQADMLHQLRLIRTNTRQQAKSDWYKWKLQWIDGLKYTAQRAFTSLQNDAKALEKLRAETDEIVPRLQREYDEVLRELEVEQQEVEEIEQCDQEYLNELKSSIAEQNVEVESLKAEVKEANDQLHWLQDRLEGVSLQKQETAGAIAEATRLLHIQTHSTQAEVWRLNNELEALEDIHMFHVIKARSNVFEYVYASEYHVVIPCRNYSPLTSQIDIVSLPEMRARFKDDLPSLSSFLLNAAKEYIRGSELSTVRQIIQRLADFWSSTTQVRTQLRQLSIKYPVEIDSRHTPDKPFTEFAVRTMVLFPAKKAKAYISFVFDLDTFSQWPVSVGSLRWEVRIGYGPIDEQAILKAVQERMKEVTPSENYACLIDACIDAQEACGVGISSSLYDVIVTRFFCPKSGETTSCNARRKVLKVGGIYLGCSPTMFRTAISRVSAISSVRAFHTSPVASKTVTEKVSEVADNVNKKLGKGLASAIEKGEGATESTKETLGSAADKSKQNTQEGVEAAKHKANQAATGASAAKEEFKKEVRK

>PSTI

MADRAELIRNAVTFLADPKTQVSPLAQRIQFLEAKGLTSAEIEEALRQASVNATAVPPRYTVYGPAYGPSPYPLVPPAAQPWDWRDYFIGAVVSGSLVYGAVALARKYLVPHLRPPTQTAYEEDRDALTAQFDAAEALLREIQAETSAVKTAVETQKERVDKATQEVEEAVKEMHDNQARARDELHEIREEINNIRDMLPKMIDKNKESQTQSLAELQQELKSLKALLLSRGPSLPSTPSALPPLSGRPSIPAWQLAGASDSVPNVQIGHRRRPHSIAPGDVLSPRARARGLAPRKSILKLATPVLDEEDATTNLGDTTSRKSFSRRVSFAATASVRLIAARKDLDSSVSSQDSPSRNEDPQPSTSGQNDENAYPGARRRSSLNGRRRMSSLGNGDEESMDLDDTFTGDLPPAFGNAGVGSALDSSDEDAEGEDGEDMEETRPMSNAAMRRRSSIGLRRAALSSKQSAGPDEGADTSQVDMSMESAENSRGDESMGSVGNESAGPMEFTVPLAQALRKPEPPSEAWLALQSATHSGINPPSDEADGDHEGGQAGGMELDDALSRLRKARDSMGFAAEEYPSPRNFLDENGGEPSFVPDDDDFSDSSFGAEDGDEGNQTLNVTALMKRRSLGRMSVGAESAMSLDDEDWQGDKIYPDLSGIEESPPAPAPAPPPAPAPFVPFRDPPALEKPPPSPGRFSAAMSRSIRVDGQIASQPEPAGMIGGGSPASRASTSGEVSATSTDLALHEKRGIFSAPGRVANPSVFSAPGTSSASEVPPRPGSALGRSVFSAPPPRATSAPPSPIKSPSKSRPSRQASAEPPPQANGPAAPTPMRYAFTPRSNKPTVSGSPSKQQAPQFSAAFAPPSARKEPGAMRRASLGQATNAENNAPKRPATEVQRPGPSKLPALSTQIPDSNVTTEQAPAEPTPALLRPSLSAAKRASFDALSATTPGASSAGPRRPSVGRAASLSGQPAIATPPQSHDTEPGPGSAQQNLSPPAPPAQQEVPPTPVRRMSALPPPSPGVWAASPGKAIRPKSPAAARSAARAQPSPSKPRRRSLAEIELEDDIEIEDPTVQWRNGVHQESLDGVENGPSISIEQFMEMTGVKFMDELTAPRRSTIRPSQLRRSLGGDTSSEEASLQDYFTAMTIDIPQLELYSNAMKQLQAGIEHSKQTFTEVNEEAKNNTPELFKEYVEADEDNKNFLLHELKLIKDNCHKVSKAQWYNWKTEWVEHLHEVAERGLSSLQADAEVLSTIVEKSSAILPNLREEYDAVMRELAEEQAAVAEIENCDQDYLEELKATIAEQNLAIEGYRADVADRESKLQRLNEKLADTRVQIQEGNSAIAEAQRVLDVQQGSTIVEVLRMQDELEFIQDIHMWRIVKVHPDYLEAVYDSCIRVAIPCRNFQPMTEHVQVSRVKDKLPVRKDWFPAYNDHVLQAAQRQVASRKAILRHSRDIIQILSDQFAVCALLRKQLHLLVTRYAYETAVAPDGSSFAVTVSLLFERVRTKALVSFTVDTETMCTWPQQAERIAVAVKVAYGSLKPENIHQILLDFLQQNHPADNPRCFSEACEEAIKSCQ

>AGOS

MEPHKRYSLPPRSILKQKSNYDDDERTSDTLQISSQIQFPTHGLTKDQLLNGNNTTSRINTTQLEAKLNRRVSFAPDVTLHKFDFIPEVPVKFREPRRKSSLSVLEKSHDNDTMELTNPVTHAFDGLGRVEEPDAGYEAVFDKEVSMEITQLFTKHSARPESEAEGAEAGLGRAPVEETMELTNLHNLHGPLNTGTGDESMEMTDTHQAGPAPNEAEKNDANNGAEEEEDGGDDAPTPATEESMEITGQQSVGRIPSSGSPSAKLSGVATARMVSMDGDSMEITGLQQLSQAHEYREAPDATLTLSNVFEGPKHLNEQRAEETMDFTAFPKLNEALTIDSQVVTSTQEPPAYKALSTIKISPKRRKIAEGIYVSPVKPNPIEELFSDAERLSPIPLPPEYSPSRQANSSNPVLSPADVPKLPHDNYAMKAVDQQPKEEPVEPISLQMFLDTTGISFVIDLDGVQNYDPITFTYTDRIEDISTRQIYDALYLQIPLLEIYAFIVKELHRRILDSQRLFQELEEQISNNPPPYLFRNYFESSDEVKQLMKEQIVLIKSFARLEAKKVWFEWRCQHLKGIKSVLEENLSLVQTEYAEVVARLNEISDIKHRLQALEQSLRHELELLRNGEKPMRVTTLADRLKIEKIKSELKANMIKLNNTANLEEQKEAISADINALRSQIKDVREEISSLKSLVLKNKLHTAHDVSKLRLLFSQMQLFTGVSFRGLSGSELQLALNSTINVSFDLTQVDNIKDIAVSYQTESQFERHISTLFINEARHAAPNAFTFVLAVQSKLQHIRNVCMEYDRLQAIFPTRLVTVRDTDLIELTDLDAVRNAKVHYHIGIPDFVAAVTSADQKKITIRARVAYGKGVTQELLERHLLSRTKKPLPWFRQFQVVL

>PCAR

MDIKSPGFKWESKKQEILNKEILNKENLSPNRSKGTVLGEGPYLKRSKSFDGRGDEKFVKPLLGKREPRSTTLEMPNKGILKTSVYSLGSAQDSSYKSDMDNHTVIGIMQVALKDNEKETENGEMKRNQGRKSLNRRVSFANYASVRLFEKEQEFQSPNPSSPVKTTTSLKENFLLSPIKRNLKTKKSTVLNDSPKTPSPIKCISPIKPSKAYINSPIPIPQGQEFSDDIDHVDIESSENVFFSDIGNNYGKGFRDCEDNGVFKDIDGEMTMDLTEVISNPVFEEKNAQEVAMDVVDVVSDVVSEEQDVEVTMDITKVLPCSILKKNVTEEPLIHLTNVFDNSVEKNNEVAMDLTVALGGIQEDQSKLLFDFQSQSDKKYTEKLAMELNTTDHGFLKIANHTDPVDMDTTKVVGGILRNLNEIDQKDGLTMDFTTTIGNILTVNEEIKKDLDFSSNNVCSEEKSDIQRKMQSDTSTECFSLDDFHKPVDVYEDDNTVGMDLTKIVPSILEHKDNLTLKNVEKEYVSLEHDSLPMGIDIKKSPNTELKSPQRIPRNGVLAMDALFGKNTLNNIRNVSDTVAPIQLGSPKAAKLLKSRKSIGCYEEMKLVGLGSPTTKIIVGDKSTRENGTSGLDIFTVNGFNILDRIAQLTPKKNVPFQLTPEKSVISSQLSLQNTSKDDIHENSESRLKRNYENTLNDADIISPMKKTCEGSNIKEYSDHLIETHDEDNFLPSITLTEFLKMTSISFLDGLTTTKRRETTFFPYLNTNPPTLKELVYASSLTLPTLELYQFSCKELQKYISEGKEVVNKIEEDTSEENPLLFREYIYASHDIRVIMDGQFKLLKNYSRLYAKSVWYDWRDKLLLGLKEGLEKNLEGLKKDELIINESKPILNTYFPFIKQTYKSLKEKVKHMKAIKEEISQCDQKELKKVRSNLFKINEELQADMKHFEKLKKNIHEIDDKLHELSEKQIKLNQEIEEYKKVIEVNRCFEKGEIENIRKMLQVLKFVTGWEAKHFFHDTVVFIYLDTIETTFNFIKNNVSIKWTGDIKDKIKEFFFDYLEKRLVGCTVKEALSLICSIWYTVTLIWSEFCLLKVRYPLLYNLTIENGEPLLLVTAKVFLPLSFSKFHVTFKLRHSILYEYPNLNNYDLIDIHTVYGNSNTSKILKAILEKISVGGIKALGSVCSDF

>TDEF

MNKENVAATPTFTRKRAKSFSTPPTTKPATSRISRGILKGNGGDDAHTIAFIPIHSPSKKTSESAEKRRKSLSRRVSFASHASVRVFEKAINSSPQTTPRRSNRLSPAKSPITDKLEDSPTRSPLAARFANNDDSNDELTMDLTSIIPKQTNPLTDALLNSDDDDESEDDFSNDEQAEDETSAMDLTDVLPVASSHASMSDVVLTPRQSRLPIRSKPSPKAQVYNDDNDETQLMDMTRVFNTEQPTSNAALQSLEDEINDDGMDMTRLVGGIISSAQARTSDNGFIASSPVKTPDNTDDGDDQTMNMDETRIFDINTIDRRQTLGTDEPTSHTSYTIATEEDDTQRLCEGSVTQADGFTQNMDITRALPTQIRTATRQSPIRQANVGFSWGANPVGEVSLMDVSMADVTQNDESVNMDMTQAIPTTFPGIYEDHSLPQEEADQDDTVNMDMTRVVASAATQLGLHSEVTLTQQSSLPLSRVSPAKTPIRQSPKKSNPSTPTAAVSTPKSASLKRRLSSGAIRTPKIALQSTSTPQKLEQARPTTPRRTPSIPRRKSLLESDIPAFGTPEAQVLLEPGSSKRPEFGSALLAGQDSRLSEMKEKIQSLTPRREVTRNSMDSPLKDRVSQTISPAKKARFTTPQKTPQRSLLAPSASVSRANSSLSMTPARAVIESPLPRTTLSEFLKMTGISFLTGLSTTRRRETIVMPEKVSESADPLDREIEHLVDQAYTVPMLEMYQHSCRELTRYITEGRAMCEEIESDMNDQNPELFDQYRRAGPAEKRDLEGKFKELKTTARLSAKGVWYVWRENLLSNVLVPLKKNLADLEKEAARLQEFEEKATPKFEQVQREYDELKAKVDELVREEDLYDSYDHEEAAKLAETIEAQEAQMAIDAAEIERLKAQDAQLDAELAAIDLEYDQKLAEVKHLEAQVEKHRGYSASEIQEMQLEYRRLVESTGVCVLHVTDSAIQLVMDDSLLVDLSLADLSVTLRLTESAADNVVQEYYITQLRQSLGEMAASSSPAAHNKIRHIRDRWTAVGRLMQELEAVESAHPLAVRVTEDDNLEATATVLLSASRTKFLVHLTSTPSLSLEDCALAVEVEVKYGGLDGAKVQRHVQARLHGQASQGRRTAGWLLESLRMPEGL

>RMIC

MSSLIPPSPSRRRRYEALKQISWTLDNAENRVTQLDASGTDETASEDDMIVEYELVDEQNYIAYQELEEDEDMQMTQEIKDDDVDMQLTQEITYNKPVKDAVVMQEETMTTNDKPSLEDSEQTTLADFLEMAGITFPRQIPLNDIIPISIVDVDYESPTIAEQAAAAAFTLPQIDMYDSISKQMHSLIDASQDVIQKVEDRVNRTSTEFFSDYLRANASTRITMEPEYRLTKQYAELKSLERWYEWCVNIFAEHLGTLKGHINTLTQDRQTLASLEDELRGQLPKIVEYQQGISQLLAEAHEIEKEYRRFDHKKLSRMRDEIEHQRQTIELFNKDLENLGAEEAELFERIDMLEHRKSRLIKDIVDAREITSVNHIITENDLNQVRQLYERSCNVGGLRLLKDNEDDDTLEILVAKCLVLTIYRNDLKERKEDAVLFHIKNNKINGYEYIAHFRRGLSAIVKSNQDTNQIIQEITTYWNRVQMLVDVLKETELRFATTIHPIETNEDIESGISCSIDVASKKRRQKIAIKFDIRVRDIVQFPFIDDSSIQVKVCFNPLMTNEIEERFKEYLNENGILELVDGITSLL

>RIRR

MEGKRELRRSPRLNKSPKRRRNSLLPPPTKSILKKSEDTTNFDILQDENTTTTNVLGSLWESNTTIFTRNFDESEITENYDGQKRRKSLGRRVSFASKARVRLFDKDEATENQEPELDKSNSLNGNKDEASEFMLSIPTPSPNDSMIENVESSSSTTNSQSEEDGKGKYAESTEKKIMISNDSQSTSQNMSQAQNSNTQMICQNNTVLGPVNDQKSFKDHDDTFASKDMSLVSMIGPTSIGRHDTTSTSVCPCDMTLVSMVNQSPSGNHEITNTSVDAQDMTLASMVGLPPSENCHTSMNVQDTTRISMANQPSSENGTSMNVRDMTLASIVGVSPSENCHTSMNVQDTTRISMVNQPSSENCTSMDVRDMTLASMVGVSPSENCHTSMNVQDTTRISMVNQPSSENCTSMDVRDMTLASMVGVSPSENCHTSMNVQDTTRISMVNQPPSENCTSIGVRDMTLTSMVGVSPSENCTSMDLRDMTIASMVDMSPSGNNTLINVKEMTLASLADQSPPKSMGDKNMTPTSMINRSPSESMGDKLTPIINQSPSKSMEFKNLTPTSMVSRSPSDFKDMTLASMVDMSSAEIHKITSTPIDVNDITTADHSIISSEDMSLVSEENVYYNDKRTIFQGLDTNLQPSERRETTLFDVTDASLINEANVKLCDGTTAMLCDENITDIMINETINTGDLNIKEYQKSDFADTEEMKFMKAMIEEQNQQIEFFEAELEGINKETQSLREMRDYLIKEKAEIEQKIRDAKRLVEGTQCYTEKDLNEVQEQYRILIDKHKWFPKKLSEELVHLVYDETVQVKIDTRDPCKRDDTNIPFVEVSLDLNDDMKYDEKLYYQAIFGGIQKFALNYKGTIEIAACSRIVKPIATYWRNSKQLYRDYRVLRFKLPTEIIDSQIINNEKSIIMMIRVNFFNYKAKTNFYIKFLFRWEDVISYPNISNLLWEIEVIYGKVNNPFIKETLNKHLSSNVLGCIRDSCVVIQKNGFVF

>SARC

MSRIPVPITQVSTAADHTASHSESFGPPVLDSTGPNPVGDGQGDAMAALFEGDNLSDVDMEETVALPIRVETFPANFNRQPSTSYQDGRATEVPENDTCNLAEEMELTKCLPSLLPQHTSTSKPNFPEFQRAPIPAPNSVSHQNPNANSGADYATTTTGASIQNPVQDSNAPASSVPVPLSVPEFTTAPTARAMAGSEDPYDHHVLNDSFEEDFGDGRADATVRMDMTELVGRGILQANGLLANEIAENSSSRSNENGAMASGDQLTANENTEFMDFTEVVGGVLSASGETGSTNANTGTNRNISTKKVHVGPNLGEQDSHIAPRGRNKVDERSVNTQVDMDTDTTNTMRMDMTEAVGGILHALPASGDHADRSELQHKQATGDTEPQSTDTDRMDMTEAVGRILNADEGTIKIPNFFLPVLVSDDVSESSGRRDTSSPPGSKRSSPRANEKTNENAENEREDVVAKRPRRESEQLVSTLNDLSVQPSGSGGVAMVTNQQLGRPDNSDRNGTTSSRPSEVARTDTHPNPANGTEPGQDTDRVVLHTDVANTHTHTPAPAHSGQDLSSHHGDGEPTEVTTHSHTQNLTHTHTHNLTTASTTGMLDVSSISHSPTTARSLNTTRVLAATQAGLLIAQSSVAMATAEPPIEATSTDQDQNTLVAMATRRGGSRLSVSRAPTPVKDLGPPHCESNQCTRLPVSLTDPFHGQFGNSKVCMSVWRYW
